# Finding maximally disconnected subnetworks with shortest path tractography

**DOI:** 10.1016/j.nicl.2019.101903

**Published:** 2019-06-18

**Authors:** Clint Greene, Matthew Cieslak, Lukas J. Volz, Lukas Hensel, Christian Grefkes, Ken Rose, Scott T. Grafton

**Affiliations:** a Signal Compression Lab, Department of Electrical and Computer Engineering, University of California, Santa Barbara, Santa Barbara, CA, USA; b Department of Psychological and Brain Sciences, University of California, Santa Barbara, Santa Barbara, CA, USA; c Department of Neurology, University of Cologne, Cologne, Germany

**Keywords:** Diffusion MRI, Tractography, Lesion symptom mapping, Disconnection, Brain injury, Spatial normalization, Brain networks, Connectomes, Disconnectome, Graphs, DW-MRI, Diffusion-weighted magnetic resonance imaging, DWI, Diffusion weighted image, DTI, Diffusion tensor imaging, HARDI, High angular resolution diffusion imaging, SyGN, Symmetric group wise normalization, FODR, Fiber orientation distribution reorientation, CSD, Constrained spherical deconvolution, GQI, Generalized q-sampling imaging, FODs, Fiber orientation distributions, ODFs, Orientation distribution functions, QA, Quantitative anisotropy, GFA, Generalized fractional anisotropy, PSFs, Point spread functions, HCP, Human connectome project, CLSM, Connectome-based lesion symptom mapping, VLSM, Voxel-based lesion symptom mapping, CST, Corticospinal tract

## Abstract

Connectome-based lesion symptom mapping (CLSM) can be used to relate disruptions of brain network connectivity with clinical measures. We present a novel method that extends current CLSM approaches by introducing a fast reliable and accurate way for computing disconnectomes, i.e. identifying damaged or lesioned connections. We introduce a new algorithm that finds the maximally disconnected subgraph containing regions and region pairs with the greatest shared connectivity loss. After normalizing a stroke patient's segmented MRI lesion into template space, probability weighted structural connectivity matrices are constructed from shortest paths found in white matter voxel graphs of 210 subjects from the Human Connectome Project. Percent connectivity loss matrices are constructed by measuring the proportion of shortest-path probability weighted connections that are lost because of an intersection with the patient's lesion. Maximally disconnected subgraphs of the overall connectivity loss matrix are then derived using a computationally fast greedy algorithm that closely approximates the exact solution. We illustrate the approach in eleven stroke patients with hemiparesis by identifying expected disconnections of the corticospinal tract (CST) with cortical sensorimotor regions. Major disconnections are found in the thalamus, basal ganglia, and inferior parietal cortex. Moreover, the size of the maximally disconnected subgraph quantifies the extent of cortical disconnection and strongly correlates with multiple clinical measures. The methods provide a fast, reliable approach for both visualizing and quantifying the disconnected portion of a patient's structural connectome based on their routine clinical MRI, without reliance on concomitant diffusion weighted imaging. The method can be extended to large databases of stroke patients, multiple sclerosis or other diseases causing focal white matter injuries helping to better characterize clinically relevant white matter lesions and to identify biomarkers for the recovery potential of individual patients.

## Highlights

• Significantly accelerated shortest path tractography approach for constructing connectomes and disconnectomes.
• New algorithm extracts the subnetwork containing cortical connections and regions with maximal shared connectivity loss.
• The size of the maximally disconnected subnetwork quantifies the extent of disconnection and correlates with stroke measures.
• Fast and accurate approach for visualizing and analyzing the disconnected portion of a patient's structural connectome.

## 1 Introduction

For over a century the relationship between lesion location and clinical deficits has been used to further our understanding of regional brain function and to predict neurological outcome, particularly after stroke (Damasio and Damasio, 1989 & Binkofski et al., 2001). Traditional voxel-based lesion symptom mapping (VLSM) has proved particularly useful in characterizing the functional specialization of discrete cortical regions (Fox, 2018). However, VLSM methods can be uninformative when symptoms are not clearly linked to the damage of a specific brain region or when the lesion extends into white matter, causing a concomitant disconnection of different cortical regions (Lim and Dong-Wha, 2015). Since human cognition and behavior typically does not arise from a single brain region but rather results from emergent activity across neural networks via interconnected cortical regions (Geschwind and Kaplan, 1962 & Baldassarre et al., 2016), the potential of VLSM to accurately explain structure-function relationships remains limited. VLSM is not suitable to characterize white matter lesions in large part because it relies on T1 weighted images which biases it towards detecting necrosis and gliosis in cortical tissue. This has led to methods that can take a network perspective that include white matter lesions (Carter et al., 2012). In particular, connectome-based lesion symptom mapping (CLSM) approaches are now integrating connectivity information based on functional or diffusion weighted imaging to improve the mapping between patient's lesions and symptoms (Yourganov et al., 2016 & Gleichgerrcht et al., 2017). Although functional connectivity based CLSM approaches have proven their clinical utility for gray matter lesions, they are unable to provide connectivity information when a lesion is restricted to white matter because of a lack of meaningful BOLD signal in white matter. Several different strategies have been developed to perform CLSM based on structural data to investigate the clinical impact of disconnections of white matter pathways. Typically, the strategy is to project the patient's lesion into a normal database of streamlines to assess how approximated normative connectivity is disrupted by a lesion (Kuceyeski et al., 2013 & Thiebaut de Schotten et al., 2011). Similarly, predefined sets of white matter tracts in the normalized space can be used to compute their intersection with individual lesions. Disconnections are typically quantified by the proportional volume of white matter tract affected by the lesion, referred to as “lesion load” (Zhu et al., 2010). Instead of calculating the volume of the lesion within a given tract one can compute the proportion of streamlines that are severed by the lesion (Hope et al., 2016). There are a number of challenges with both strategies. Streamlines are a computational construct that are extremely sensitive to arbitrary parameter choices such as angle cutoff, step length and total number of streamlines generated, making it difficult to compare results (Wei et al., 2018). Furthermore, they are known to suffer from a hard tradeoff between the detection of true connections and the generation of excessive false connections, particularly for crossing fibers (Maier-Hein et al., 2017). Compounding the limits of this method, volumetric measures such as lesion load can be misleading because a major tract such as the corticospinal tract can be severed by a very small lesion in the posterior limb of the internal capsule resulting in a severe hemiparesis, yet a majority of its volume remains intact. Moreover, studies incorporating the alternative method: percent of tract loss, either quantify the lesion impact at the level of individual regions or simply provide a binary measure of whether the tract was severed (Kuceyeski et al., 2013 & Thiebaut de Schotten et al., 2011). Only recently, researchers have begun to quantify the relative amount of damage to a given fiber tract connecting region pairs. Using such an approach on DTI data in a large sample of patients, Langen and colleagues found that cognitive impairment was associated with disconnectivity, i.e., patients with higher percentages of damaged streamlines also suffered from pronounced cognitive impairment (Langen et al., 2018). While such quantitative analyses of streamline damage represent a promising approach, disconnectomes are constructed in the patients' native DTI space which can produce distorted disconnectomes because the diffusion information is corrupted in lesioned white matter tissue (Maillard et al., 2011; de Groot et al., 2013; Theaud et al., 2017). Tracking through regions where orientation maxima are distorted produces distorted connectomes and consequently distorted disconnectomes (Greene et al., 2018). To overcome this limitation, we here present a fast and accurate approach for estimating connectomes and disconnectomes that does not rely on streamline tractography and avoids tracking through lesioned white matter tissue. The approach embeds a patient's lesion, segmented from their standard clinical MRI data into a normative fiber orientation diffusion database. As such, the method allows one to assess disconnection without the need for adjunct diffusion imaging and therefore can be used in a standard clinical setting.

Accurately measuring disconnectomes at the region to region level with streamline based tractography is difficult if not impossible for many region pairs due to systematic biases in estimating long-range connections (Li et al., 2012; Sinke et al., 2018). As an alternative we construct connectivity matrices using whole brain shortest path tractography where region to region connectivity is weighted by shortest path probabilities. Specifically, we use data from the Human Connectome Project (HCP) to reconstruct each subject's fiber orientation distributions (FODs) using constrained spherical deconvolution and normalized them using FOD reorientation to a custom high-resolution template (Raffelt et al., 2012). For each HCP subject a white matter voxel graph is constructed in the template space using analytic tractography, which obviates the need for lengthy probabilistic tractography simulations (Cieslak et al., 2017). The output of analytic tractography at each white matter voxel is a 26-element vector containing the negative log of the probabilities that a white matter structure transitions into each of its neighboring voxels. White matter voxels located at the gray-white boundary are defined as interface nodes. These are partitioned into cortical regions using the Lausanne atlas. The shortest paths and their probabilities can be calculated for all pairs of interface nodes in different regions to capture region to region probability weighted structural connectivity. However, constructing shortest-path weighted structural networks from all pairings of the interface nodes is computationally costly because there are nearly 2,000,000,000 possible shortest paths per brain. To expedite these computations, we show that structural networks constructed from all possible pairs can be rapidly and almost perfectly estimated by uniformly sampling subsets of the interface nodes for a given region pair. After the anatomical scans from stroke patients are normalized into our template (along with their segmented lesion), the percent loss connectivity matrix or disconnectome can be obtained by querying all shortest paths that intersect the lesion and by computing the proportion of shortest path probabilities that intersect with the lesion and hence can be assumed to be lost.

Given a particular patient's lesion size and location, the full disconnectome can be quite extensive, making visualization and clinical correlation challenging. With that in mind, we describe a new graph theoretic algorithm that quantifies the extent of cortical disconnection and reduces the dimensionality by extracting the maximally disconnected subgraph containing the regions with the greatest shared disconnectivity due to the lesion from the disconnectome or percent connectivity loss matrix. Although thresholding can be used to reduce the size of the disconnectome, it is an arbitrary edge based approach that produces subgraphs with no guarantee of shared disconnectivity due to a lesion. Our algorithm produces a clinically relevant disconnection subgraph that makes visualization tenable and produces a remarkably reliable estimate of the number of cortical regions (*k*_*optimal*_) making up the disconnection subgraph.

## 2 Methods

### 2.1 Normal database

The S500 dataset was collected from the Washington University-Minnesota Consortium Human Connectome Project (Glasser et al., 2013). Further analysis was restricted to 210 subjects without familial relation. The data consisted of structural and diffusion scans corrected for geometric, eddy current, and motion distortions. The diffusion volumes were collected with a spatial resolution 1.25 *×* 1.25 *×* 1.25 mm, using three shells at b = 1000, 2000, and 3000 s/mm2 with 90 diffusion directions per shell and 10 additional b0s per shell. High-resolution structural T1w and T2w volumes for each subject were acquired on the same scanner at 0.7 mm isotropic resolution. Generalized fractional anisotropy (GFA) volumes for each subject were extracted from their generalized q-sampling imaging reconstructed HARDI data in DSI Studio (Yeh et al., 2010) for subsequent multimodal image registration.

### 2.2 Stroke database

Eleven stroke patients (mean age: 62.0 years ±9.3 standard deviation; 9 male; 9 right-handed) suffering from a first-ever ischemic stroke causing a unilateral hand motor deficit were recruited from the University Hospital of Cologne, Department of Neurology. Inclusion criteria were: 1) age 40–90 years; 2) ischemic stroke as verified by diffusion-weighted magnetic resonance imaging (DWI); 3) unilateral hand motor deficit; 4) no other neurological disease.

Exclusion criteria were: 1) any contraindication to MRI (e.g., cardiac pacemaker); 2) infarcts in multiple territories; and 3) hemorrhagic stroke. This study was approved by the local ethics committee and all subjects provided informed written consent.

### 2.3 Multimodal template construction

Previously skull stripped, aligned, and distortion corrected T1w and T2w volumes were obtained for each subject and then rigidly registered to the subject's GFA volume. ANTs symmetric group wise normalization (SyGN) method was used to construct a custom multimodal population specific brain template from 40 HCP subjects chosen at random from the larger dataset, using 5 iterations (Avants et al., 2010). All subjects were spatially normalized to this custom template using multimodal registration in ANTs. Freesurfer was used to segment gray matter from white matter and CSF and to build surfaces of the template brain (Dale et al., 1999). The Connectome mapper toolkit was used to parcellate the cortical regions based on the Lausanne 60 atlas (Daducci et al., 2012).

### 2.4 Diffusion reconstruction

The HARDI HCP datasets were reconstructed using constrained spherical deconvolution (CSD) with a maximum harmonic order of 8 (Tournier et al., 2007). The largest b-value shell was used during reconstruction. Using the software MRtrix, fiber orientation distributions (FODs) for each HCP subject were generated using CSD from their diffusion data (Tournier et al., 2012). The FODs were reoriented/warped to the multimodal template using apodized point spread functions based on the ANTs output from each subject's symmetric T1/T2/GFA diffeomorphic registration to the custom multi-modal template. Specifically, each FOD is decomposed into a series of weighted spherical harmonic PSFs. The amplitude of the negative lobes of the PSFs are reduced, then each PSF is reoriented using the local affine transformation estimated from the Jacobian of the total deformation field, and finally recombined into the full reoriented FOD (Raffelt et al., 2012).

### 2.5 Voxel graphs and shortest paths

White matter voxel graphs were constructed for each HCP subject using the double-ODF method in MITTENS (Cieslak et al., 2017). To do this, transition probabilities between each pair of adjacent voxels (whether a face, edge or corner) are calculated with a closed form analytic solution. Each white matter voxel where the FOD is nonzero is treated as a node in a graph. Edges are formed to each voxel's 26 spatial neighbors, weighted by the transition probabilities calculated by MITTENS i.e. the probability that a white matter structure from the source voxel continues into its neighbor. The voxel graph was restricted to only white matter voxels using a white matter mask from the multimodal template to restrict shortest paths from entering gray matter or cerebrospinal fluid. The shortest path between any two voxels can be efficiently found using Dijkstra's algorithm. It corresponds to the globally optimal path that maximizes the product of all the probabilities at each edge making up the path. The shortest path is assigned a weight by taking the geometric mean of the product of the probabilities for each edge making up the path.

(1) $$w_{\mathrm{path}}={\left(\prod_{i=1}^{n}p_{i}\right)}^{\frac{1}{n}},\text{where}\;n\;\text{is the number of edges in the path}$$

### 2.6 Subsampling

The complete gray-white interface consists of 64,109 white matter voxels in our template. To find a shortest path between every possible pair of these white matter voxels requires nearly 2,000,000,000 calls to the Dijkstra algorithm per subject. On a 120 compute node cluster, it takes ~24 h to calculate all possible pairs of shortest paths, making this computation prohibitive for the full HCP dataset. To speed up the calculation of shortest path probabilities by orders of magnitude, we uniformly sample subsets of the interface voxel pairs.

**Fig. 1:**
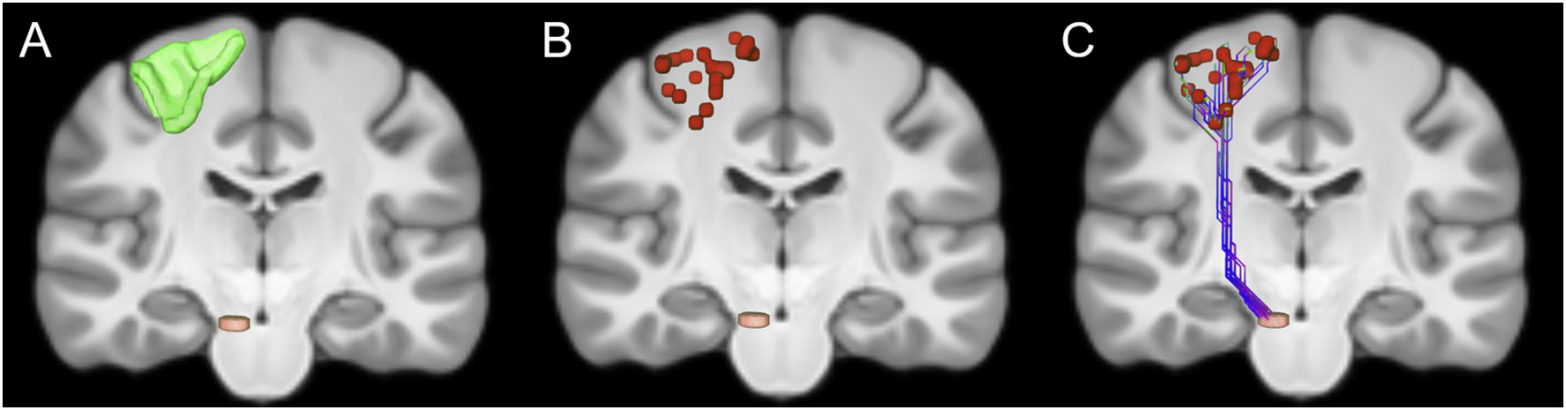
Illustration of subsampling. (A) The white matter surface of a portion of the left precentral region (green) containing 844 voxels is plotted with a portion of the brainstem (orange) containing 30 voxels. If all possible pairs of shortest paths were found between the regions there would be (844 × 30) = 25,320 shortest paths which would be a lengthy computation. The computation can be significantly sped up by employing a subsampling approach where the larger region is uniformly subsampled to obtain a subset of voxels such that the number of voxels in the subset matches the number of voxels in the smaller region. (B) 30 voxels subsampled from the larger precentral region are plotted in red. Every voxel in the subset is uniquely paired to a voxel in the smaller region, producing here 30 unique source-target pairings for shortest paths queries. (C) The 30 shortest paths found between voxels in the brainstem and precentral area trace out the CST. This procedure is performed for each normal HCP subject to generate unique sets of pairings and shortest paths for any given cortical region pair.

Given cortical region A with *m* interface voxels and cortical region B with *n* interface voxels, there would be *m* *×* *n* shortest paths if each one was found for every possible pair which would be a lengthy computation typically involving tens of thousands of shortest paths. The computation can be significantly accelerated by employing a subsampling approach where the larger region is uniformly sampled to obtain a subset of voxels such that the number of voxels in the subset matches the number of voxels in the smaller region. Each voxel in the larger region is paired uniquely to a voxel in the smaller region producing a total of min*(m,n)* source-target pairings and hence shortest paths. This is performed for each normal HCP subject for each cortical region pair to generate unique sets of shortest paths. The subsampling approach is illustrated in Fig. 1. In the Appendix, we validate this approach by measuring the similarity between structural networks constructed from all possible pairs of shortest paths and the subsampled shortest paths. Our results in the Appendix show that subsampling is a fast and accurate approach for studying brain connectivity.

### 2.7 Connectome and connectivity loss

The Lausanne60 cortical parcellation was projected onto white matter voxels lying at the gray white interface. Note the method is flexible and any cortical parcellation could be projected. A shortest path probability weighted structural connectome is then constructed by computing the shortest path from every uniformly sampled subset of interface voxel pairs in different cortical regions for each HCP subject. To compute the percent loss of connectivity, *L*, for a cortical region pair, the cumulative weight of the shortest paths that intersect the lesion, *W*_*in*_, is divided by *W*_*total*_, the cumulative weight of all shortest paths between the two regions to obtain the weighted fraction of potentially damaged shortest path connections. All weights are estimated using (1).

(2) $$L=\frac{W_{\mathrm{in}}}{W_{\mathrm{total}}}\;$$

**Fig. 2:**
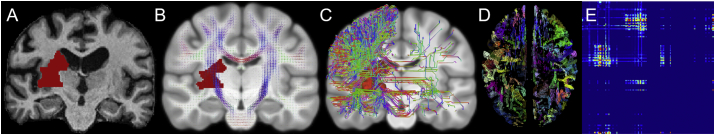
Schematic of patient disconnectome construction. (A) Lesioned tissue (red) is segmented on the patients' T1 weighted volume and (B) normalized into our high resolution T1 weighted template. (C) Shortest paths that intersect the patients' normalized lesion are found. (D) Region labels are then assigned to the end points of each of the shortest paths. For each shortest path making up a region pair, the probability of that shortest path is calculated and added to the running total of connective probability lost due to the lesion for that region pair. (E) The disconnectome is then computed as the fraction of connective probability loss relative to the total connectivity probability shared for any given region pair. (C)—(E) are performed 210 times for each set of shortest paths from each of the normal HCP subjects. The final connectivity loss matrix, L_stroke_, is taken as the average of the 210 disconnectomes.

The connectivity loss matrix *L*_*stroke*_ for a given stroke patient is taken as the average of all the loss matrices *L* calculated using each HCP subject's shortest paths (as defined by that stroke patient's lesion). The procedure for calculating the connectivity loss matrix *L*_*stroke*_ is summarized in Fig. 2.

### 2.8 Maximally disconnected subgraphs

In graph theory, this problem is most similar to the heaviest *k*-subgraph problem where an undirected weighted graph is given, and the goal is to find a subgraph with *k* nodes with maximum total edge weight. This problem is NP-hard but can be well approximated using a greedy approach (Ravi et al., 1994). Given a disconnectome, such an algorithm would extract a subgraph of *k* nodes whose edges have the greatest disconnection. In our algorithm, we use a greedy approach similar to (Ravi et al., 1994) and extend it by automatically finding an optimal number of nodes, *k*_*optimal*_, to grow the heaviest subgraph. The growth profile of the magnitude of the change in the weight of the subgraph for each *k*th node added has a concave shape with a clearly defined maximum. The magnitude at the maximum and the numbers nodes *k* it occurs at we define as *ΔW*_*optimal*_ and *k*_*optimal*_.

To find the maximally disconnected subgraph *D*_*max*_, the following approach is used. The disconnected subgraph *D* is initialized with the nodes from the heaviest edge *e*_*max*_, from the connectivity loss matrix L. In the case of a tie, the nodes in the edge containing the greatest cumulative sum of weighted node degree are added. Each iteration *k* subsequently chooses a new node, *n*_*max*_ ∈ *L* − *D*_*k*−1_, to add to the subgraph such that the sum of the edge weights to the nodes currently in *D*_*k−1*_ is maximized. The change in the weight *ΔW*_*k*_ (from *D*_*k−1*_ to *D*_*k*_) is stored in P, the disconnection profile, and *D*_*k*_ is stored in S for each iteration. *D* is grown until all nodes and edges from *L* have been added. After *D* is finished growing, a cubic spline is fit to the disconnection profile in *P* to find *k*_*optimal*_ and *D*_*max*_ is returned from the *k*_*optimal*_ element in S. The procedure is summarized in Algorithm 1, where *W*_*pre*_ is the weight of the subgraph at *D*_*k−1*_ at the *k* *−* *1* iteration and *W*_*post*_ is the weight of the subgraph at *D*_*k*_ at the *k*th iteration, *w*(*n*_*i*_*L*−*D*__, *n*_*j*_*D*__) is the weight of the edge between node *i* and node *j,* and *e*_*i*_ is edge *i* in the subgraph *D*.

Algorithm1.Maximally disconnected subgraph

**Figure:**
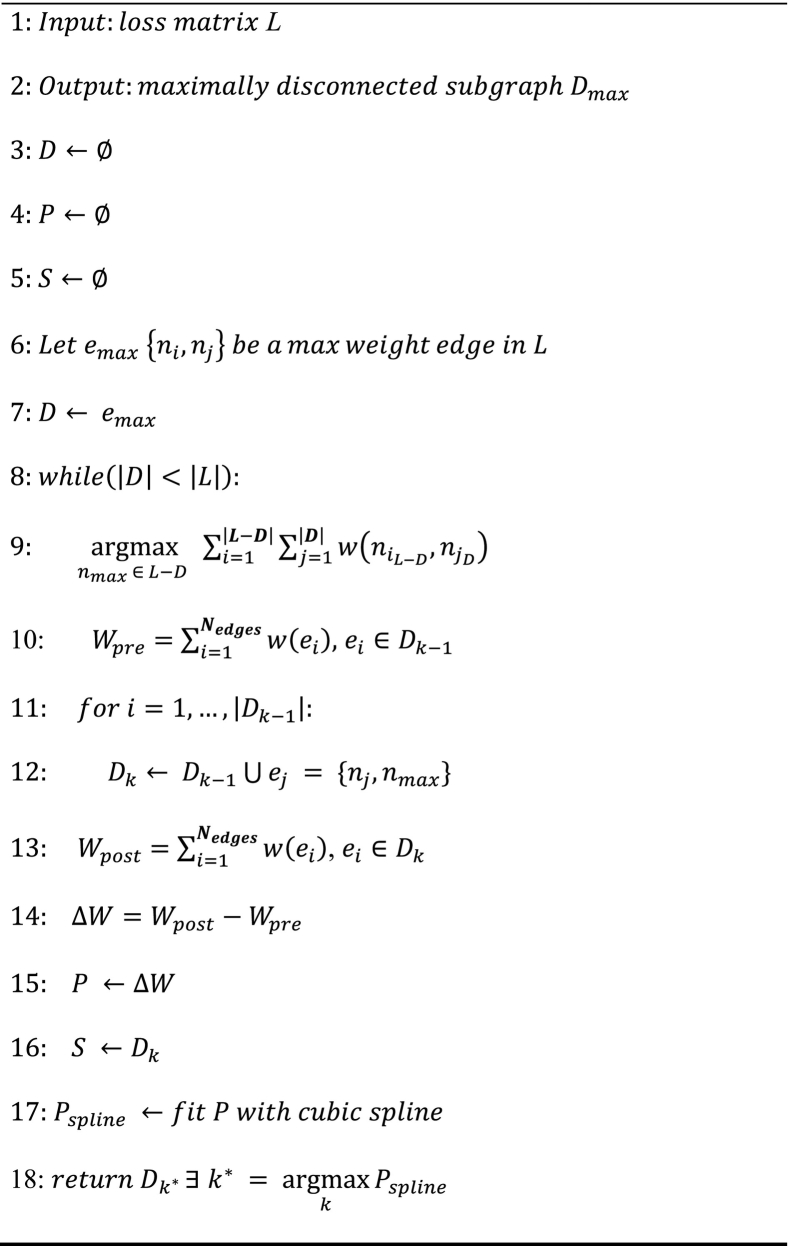

In the appendix, we demonstrate that the greedy algorithm for identifying the maximally disconnected subgraph closely approximates the exact solution. Specifically, the correlation between the change in the weight of the subgraphs for each method has an *R* of 0.99. Although this doesn't represent the similarity of the set of nodes in the subgraphs at each iteration of *k*, it shows that our algorithm is accurately approximating the *k*-heaviest subgraph. More specifically, in the appendix we show that the dice score for each iteration of *k* initially shows poor agreement between the exact solution and our greedy one because there is minor disagreement initially about what node should be added to make the subgraph as heavy as possible i.e. most disconnected. As *k* increases the greedy solution converges with the exact one with dice scores of 1.0. Moreover, our greedy algorithm is rapid, taking only seconds to find the maximally disconnected subgraph versus weeks to find the exact solution (combinatorial).

### 2.9 Disconnection growth profiles

To determine the optimal size of a disconnection subgraph for a given lesion, a population estimate from the HCP dataset is performed. To do this the change in the magnitude of each patient's subgraph as each node *k* is added from each of their connectivity loss matrix is determined first. These 210 growth profiles are then averaged and fit with a smooth cubic spline to get a robust global estimate of *k*_*optimal*_ because estimates from single HCP subjects can be noisy and the average has minor perturbations near the peak. The *k*_*optimal,*_ determined from the averaged disconnection growth profiles, is then applied to the mean of the 210 HCP connectivity loss matrices to extract the final maximally disconnected subgraph. This process is performed separately for each patient's lesion. In the Appendix, we demonstrate that these growth profiles and maximally disconnected subgraphs are nearly identical for shortest path measures across all gray-white matter interface nodes and for the computationally faster subsampled set of interface nodes.

### 2.10 Reliability

To measure the reliability of *k*_*optimal*_, the mean and its uncertainty are estimated by bootstrapping. Each sampling distribution of *k*_*optimal*_ is constructed by randomly sampling with replacement N disconnection profiles 10,000 times where N ranges between 2 and 209 subjects. For each bootstrap sample in each distribution, the disconnection profiles are averaged and the sample *k*_*optimal*_ is recorded from the cubic spline fit. For each sample distribution, the standard deviation and mean of the sample *k*_*optimal*_ are recorded.

## 3 Results

### 3.1 Connectivity loss

**Fig. 3:**
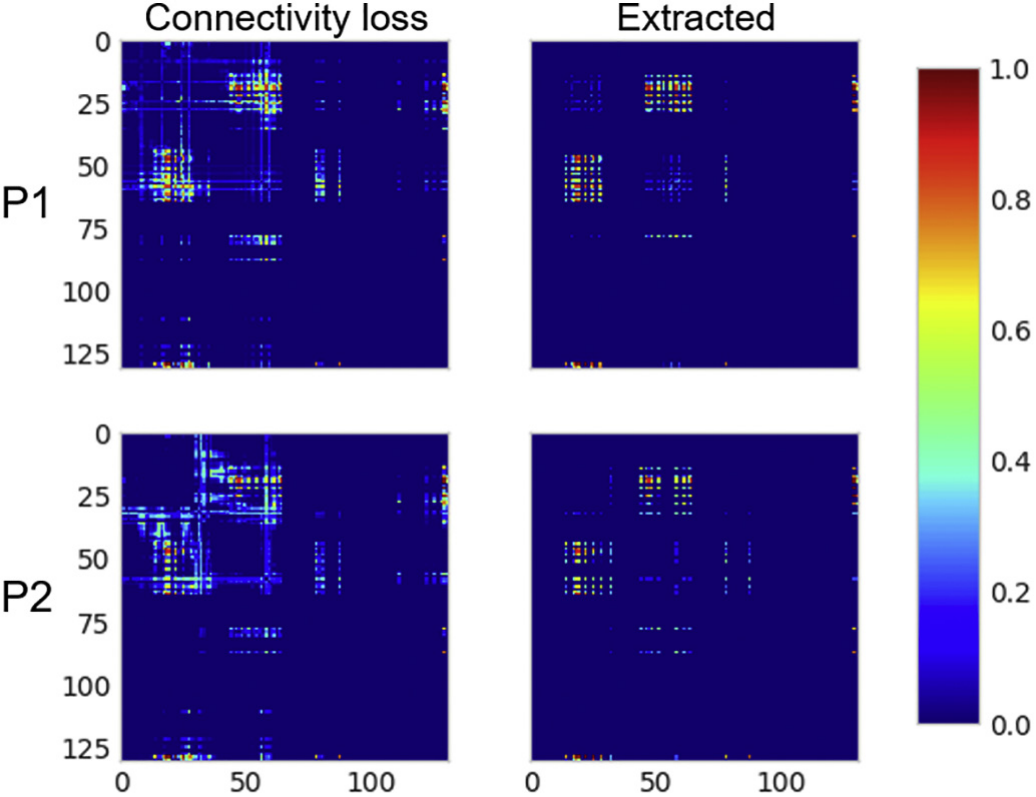
Quantitative metrics of structural disconnection. A disconnectome (Connectivity loss, left column) is shown for two patients (P1, P2). Connections in dark red exhibit 100% connectivity loss. Regions connections in dark blue were not impacted by the lesion. The set of connections extracted by our algorithm from the disconnectome forms the maximally disconnected subgraph (Extracted, right column). It consists of those connections between regions that exhibit a larger proportion of connectivity loss due to the lesion. Both patients have posterior limb-internal capsule lesions and remarkably similar disconnection subgraphs.

Our method creates a normative database of connection probabilities, based on shortest paths between all region pairs for a given atlas. In this case, there are 130 regions in the Lausanne60 atlas for 210 normal HCP subjects. Then, a spatially normalized lesion from an individual stroke patient is projected into this database and the percentage reduction, i.e. loss of connection for each region pair is calculated. Examples of the connectivity loss matrix for two stroke patients (P1, P2) are shown in the left column of Fig. 3. Each of the plotted connectivity loss matrices represents a mean of the 210 individual connectivity loss matrices defined by intersecting the stroke patient's lesion with each of the HCP subjects subsampled shortest paths. For both patients shown in Fig. 3, the lesions involve the posterior limb of internal capsule and adjacent thalamus. Not surprisingly, the two loss matrices have obvious structural similarity, yet each contains additional specific disconnections unique to each patient's lesion. Note the large variability in the magnitude of disconnection of individual connections, with some region pairs having 100% disconnection (dark red) while other region-pair connections remaining untouched by the lesion (dark blue), demonstrating the sensitivity of the % loss metric.

### 3.2 Optimal k and the maximal disconnection subgraph

**Fig. 4:**
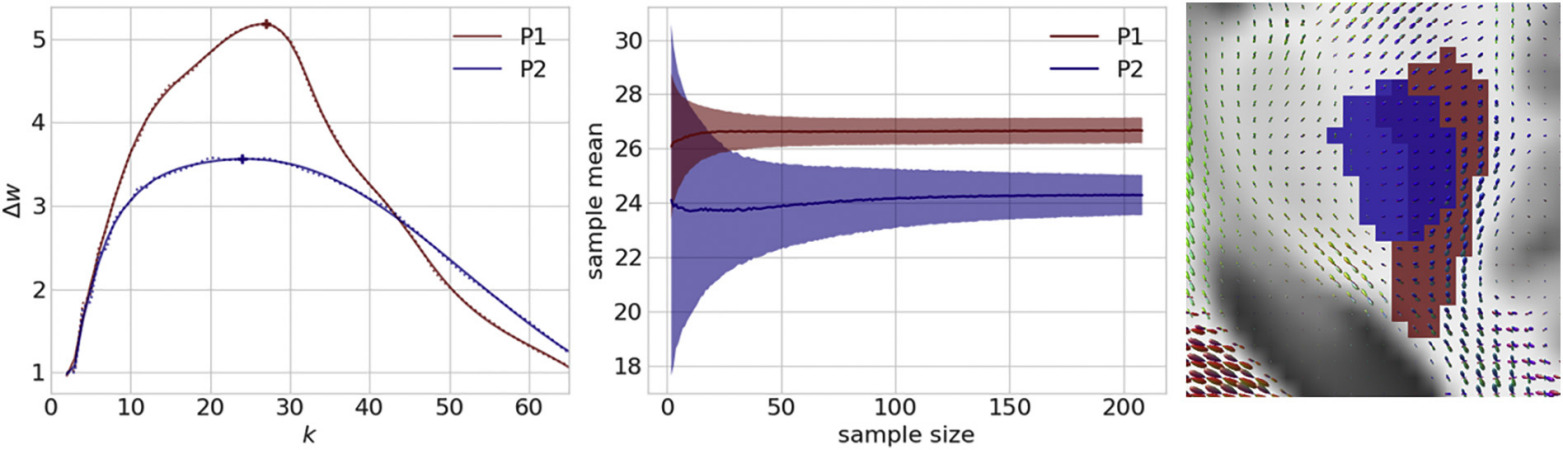
An algorithm to grow a maximal disconnection subgraph. On the left panel the average magnitude of the change in the total edge weight of the subgraph as it grows in the number of nodes *k* from 210 HCP subjects. In both patients a clear concave disconnection growth profile emerges, where initially the magnitude of the change grows rapidly until reaching the peak at *k*_*optimal*_. Patient 1 peaks at *k = 27* (red cross) and patient 2 peaks at *k = 24* (blue cross). After the peak, the change in total edge weight slowly drops off as additional nodes are added to the subgraph. In the middle panel, the reliability of *k*_*optimal*_ is estimated. The standard deviation (shaded) around the mean drops exponentially as the number of subjects increases. P1's deviation curve drops quicker than P2's. With 100 or more subjects the sample mean converges with a standard deviation ≤1 of the *k*_*optimal*_ estimated from 210 subjects. On the right the two patients normalized lesions are plotted on top of the HCP template brain (with average FODs). While both patient's lesions are nearly identical in size and in the right hemisphere, the profile for the first patient (red) peaks higher than the second blue) because their lesion extends across a more complex set of local diffusion directions where it intersects a larger number of paths.

Connectivity loss matrices from each of the 210 HCP subjects, (whose averages for two patients are as shown in the left column of Fig. 3) are entered into our greedy algorithm to extract a set of maximally disconnected subgraphs (extracted set of connections shown in the right column of Fig. 3). Constructing this *k*-heaviest subgraph is a well-defined problem given the parameter *k* of how many nodes the subgraph should contain. However, it's not clear which *k* is optimal to stop growing the heaviest subgraph. In the left panel of Fig. 4, we plot the cubic spline fit of the magnitude of the change in the total edge weight of the subgraphs for the two patients as functions of each node *k* that is added in solid along with the average in dots. This plot is generated from the mean of individual growth profiles obtained across all 210 HCP connectivity loss matrices. Thus, it reflects an average of the change in the total edge weight estimated for each disconnection subgraph of the 210 HCP subjects. This is done separately for each patient. As shown in Fig. 4 (demarcated with a +) the magnitude of the change in the total edge weight grows with *k* until reaching its maximum at *k*_*optimal*_ and then falls off slowly as additional nodes are added to the subgraph. This concave disconnection growth profile is present in all patients. Consequently, the peak of the disconnection growth profile lends itself as a natural stopping criterion and defines *k*_*optimal*_*.* For the first (P1, red) it peaks later at *k = 27* compared to the second (P2, blue) at *k = 24*. The spline fit is necessary to ensure that the global maxima are selected because there can be minor perturbations that lead to local maxima being selected. This is most visible for Patient 2's raw average plotted as the blue dotted line with minute perturbations on the left and right of the global maximum (blue cross) that are slightly higher. In the middle panel, we assess the reliability of *k*_*optimal*_ by bootstrapping 10,000 randomly sampled disconnection profiles from *N* subjects. As the number of subjects *N* used to estimate *k*_*optimal*_ increases, the deviation (shaded) around the sample mean decreases exponentially until plateauing. The deviation around P1's mean drops quicker than P2's. For both patients, the error converges to a deviation of ≤1 of the *k*_*optimal*_ estimated from the mean of 210 subjects with 100 or more subjects. Mean deviation from the sample mean for *N = 105* subjects across all 11 patients in the bootstrap with is 0.66 with *σ =* 0.38. The two patients' normalized lesions are plotted onto our custom HCP template brain (with average FODs also shown) on the right panel of Fig. 4. Both stroke patients' lesions on the right in Fig. 4 were roughly the same size containing 592 and 586 voxels respectively and both were in the right hemisphere. Patient P1's lesion (red) extends beyond the posterior limb of the internal capsule where it encounters a larger number of paths and incorporates voxels with FODs describing connectivity in additional directions besides the capsule. In contrast, Patient P2's lesion is located almost entirely within the poster limb of the internal capsule. This subtle difference in lesion shape has a direct impact on the disconnectome and is captured by the larger *k*_*optimal*_ for the first patient.

### 3.3 Maximally disconnected subgraphs

**Fig. 5:**
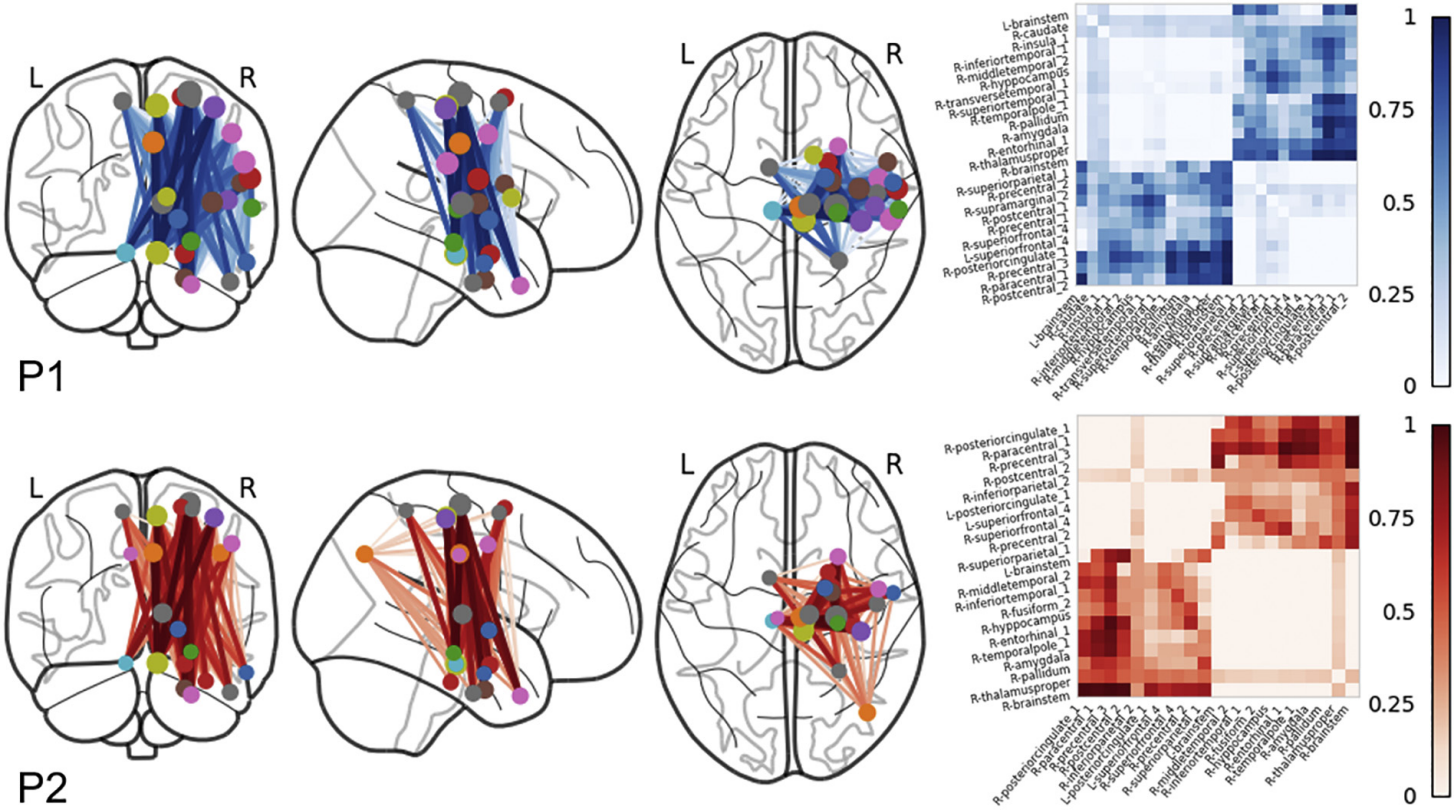
Maximally disconnected subnetworks plotted on top of the glass brain along with the corresponding maximal disconnection matrices. Region ROIs are plotted as circles with the diameter reflecting the weighted degree of percent connectivity loss. The thicker the edge and the darker the color the greater the percent connectivity loss between the connecting regions. Major connectivity loss was found amongst pairs of precentral, postcentral, brainstem, pallidum, and thalamus in both patients (P1, P2). Most of the patients' identified disconnected regions are in the right hemisphere where the lesion occurred.

Our algorithm automatically determines a unique *k*_*optimal*_ for each patient and returns the maximally disconnected subgraph. In Fig. 5, the extracted maximally disconnected subnetworks are plotted on top of the glass brain along with their corresponding maximally disconnected matrices (Abraham et al., 2014). Region ROIs are plotted as circles with the diameter reflecting the weighted degree of that region's total percent connectivity loss. Edge thickness and color represent percent connectivity loss between the connecting nodes, where the darker the color and thicker the line the more disconnected the two regions. For the first patient, major disconnections are found amongst pairs of precentral, postcentral, brainstem, pallidum, and temporal cortex. Similarly, in the second patient, precentral, postcentral, brainstem, pallidum, and thalamus comprise the most disconnected regions and region pairs. Both patients maximally disconnected subnetworks are mostly restricted to cortical regions in the right hemisphere, matching the hemisphere where the stroke occurred and demonstrating the specificity of our approach.

### 3.4 Disconnected core

**Fig. 6:**
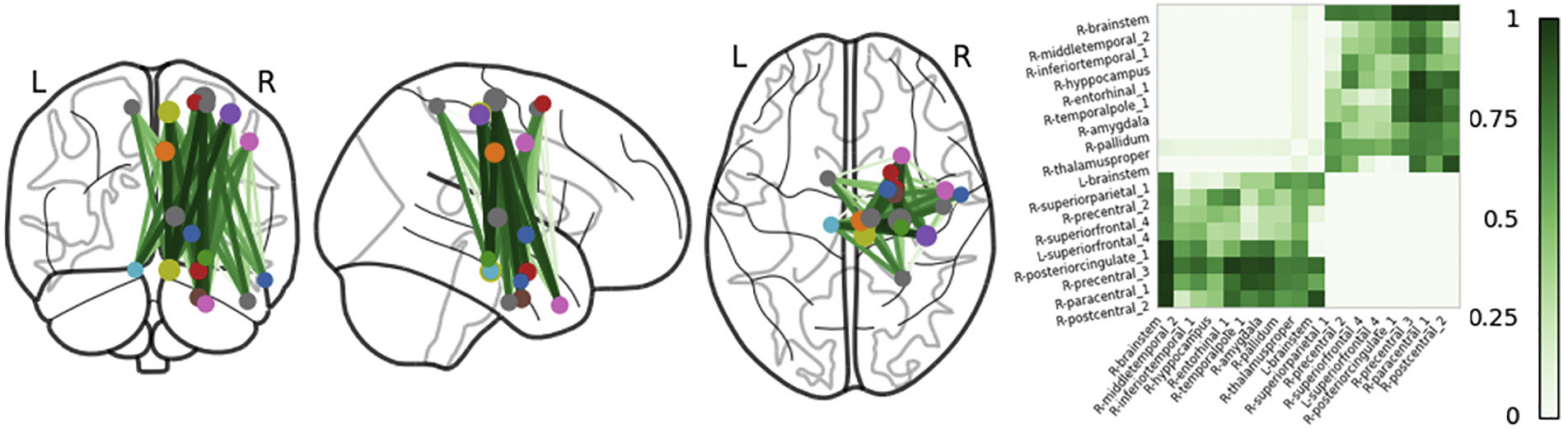
The disconnected core network is found by intersecting the two maximally disconnected subgraphs of the two stroke patients. The core is primarily comprised of brain regions in the right hemisphere involved in motor function with large percentages of connectivity loss amongst precentral, postcentral, superiorfrontal, brainstem, thalamus, and pallidum. Edge weights were taken as the average between the two patients for a given edge. This overlap in sensorimotor circuits is consistent with the fact that the patients were both selected because of similar clinical features of hemiparesis.

Just as it is possible to identify maximal lesion overlap from structural MRI scans to find regions that are most commonly impacted in a cohort of patients, it is also possible to identify a core pattern of disconnection across overlapping subgraphs. To demonstrate this, consider the disconnected networks of both stroke patients in Fig. 5. It is evident that many of same regions and pairs are disconnected. By intersecting the two maximally disconnected subgraphs, we can visualize the disconnected core network from stroke lesions that result in hemiparesis. In Fig. 6, the maximally disconnected core sits on top of the right motor network and is dominated by disconnections amongst motor regions: precentral, postcentral, superiorfrontal, brainstem, thalamus, pallidum, and caudate. The weight of the edges making up the core were found by averaging the edge weights of the two patients.

### 3.5 Dimensionality reduction

**Fig. 7:**
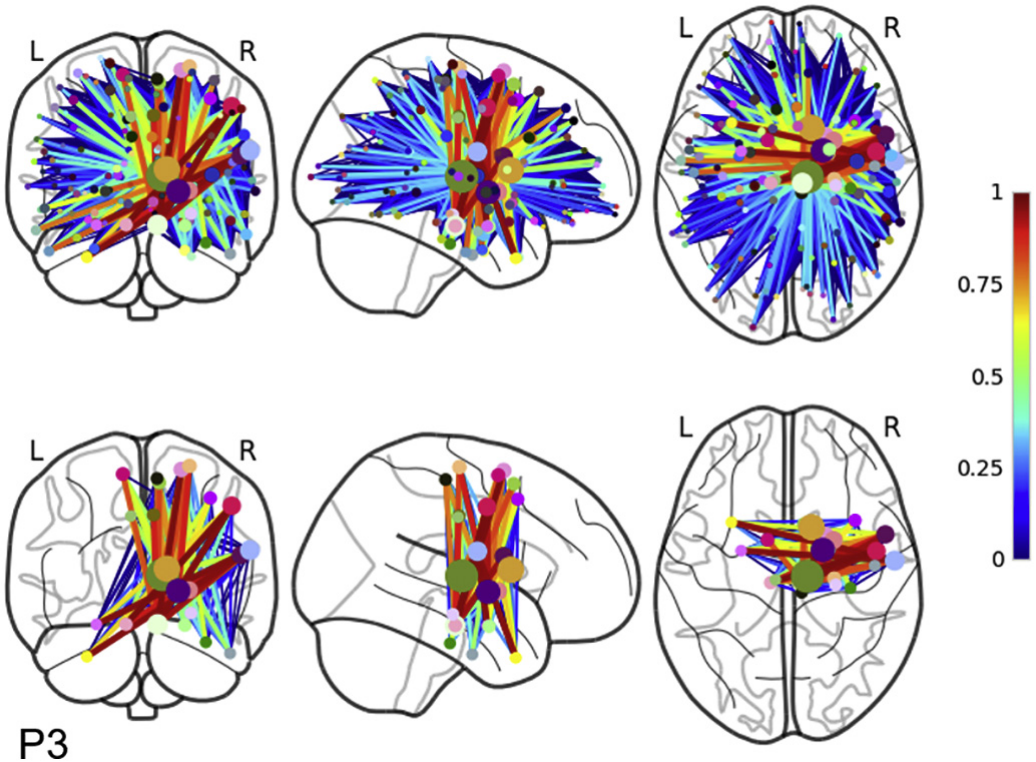
The full connectivity loss matrix plotted on the glass brain in the top panel. Many of the connections between cortical regions have a small loss in connectivity (blue) with only a small subset having a large or complete connectivity loss (orange—red). Our algorithm extracts the maximally disconnected subgraph filtering out connections and cortical regions with small losses of connectivity. The set of connections and cortical regions experiencing large shared connectivity losses are plotted in the bottom panel on the glass brain. Most of these remaining connections are in the right hemisphere and cluster around the lesion.

Our algorithm reduces the dimensionality of the connectivity loss matrix from *N × N* to *k*_*optimal*_ *× k*_*optimal*_. The size reduction is evident in comparing the top and bottom panels of Fig. 7. In the top panel, patient 3's full connectivity loss matrix is plotted on top of the glass brain. Note that the full disconnectome is dominated by a majority of small percent losses (blue) of connectivity for many region pairs with a small set of connections showing large percent losses (orange—red). In the bottom panel, the set of cortical regions and connections making up the maximally disconnected subgraph are plotted on the glass brain. Our algorithm filters out most connections and cortical regions that have small losses and are not likely disconnected (blue) and preserves the subnetwork of cortical regions and connections that share the greatest connectivity loss due to the lesion (orange—red). Also note that patient 3's lesion is in the right hemisphere and that most of the nodes and connections filtered from the disconnectome are in the left hemisphere and that the preserved connections tend to cluster around the lesion.

### 3.6 Location effects

**Fig. 8:**
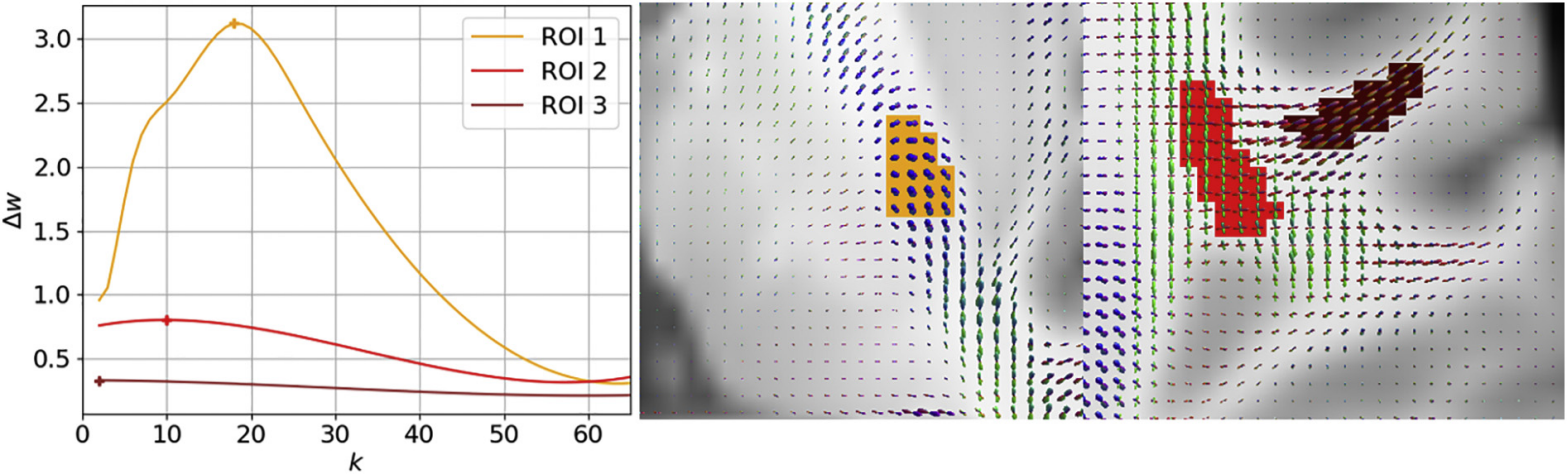
The impact of lesion location on subgraph size, independent of lesion size. On the left the magnitude of the change in the total edge weight of the subgraph as it grows in the number of nodes *k* is plotted for each simulated lesion. On the right, are the corresponding simulated lesion ROIs plotted on our HCP template brain. All the ROIs are 74 voxels in size. ROI 1 has an identical profile to the curves in Fig. 4 from patients' lesions and peaks at *k = 18* (orange cross). ROI 2 and ROI 3 do not rise rapidly but slowly drop off in total edge weight once they reach their peaks at *k = 10* (red cross) and *k = 2* (brown cross) respectively. ROI 1 and ROI 3 are comprised of single population fibers while ROI 2 is comprised of a crossing fiber population.

To further demonstrate the interplay between lesion location, the maximum of the magnitude of the change in the total edge weight of the subgraph, and *k*_*optimal*_, lesions of identical size are simulated in multiple locations for single fiber populations and a crossing fiber population in central and peripheral regions of the brain. The magnitude of the change in total edge weight of the subgraph as it grows to *k* nodes is plotted on the left in Fig. 8 for each lesion. The corresponding lesions are plotted on the template brain on the right. ROI 1 (orange) and ROI 3 (brown) are both comprised of single population fibers while ROI 2 (red) contains multiple fiber populations. ROI 1 occurs centrally in the poster limb of the internal capsule while ROI 2 and ROI 3 occur peripherally near cortex. ROI 1 has the same profile as seen in the patients' lesions in Fig. 4 while ROI 2 and ROI 3 do not rapidly rise or drop off in the magnitude of the change in total edge weight. ROI 1 has a much larger peak and *k*_*optimal*_. ROI 2 and ROI 3 both have much smaller peaks and occur earlier.

### 3.7 Relation to clinical measures

As a simple demonstration of the potential utility of *k*_*optimal*_ (the size of the maximally disconnected subgraph) as a metric with clinical use, we correlated it with several standardized assessments of eleven stroke patients' clinical status on hospital admission. We tested both the NIH Stroke Score (NIHSS), an overall measure of functional status and a more specific measure, the patient's relative grip strength denoted as the maximum grip strength of the paretic hand relative to the maximum grip strength of the unaffected hand. As has been shown previously, overall lesion size correlated with the patients' overall functional status as measured by the NIHSS (Table 1). Similarly, *k*_*optimal*_ showed a strong and significant correlation with the overall functional status. The more specific functional measure of relative grip strength, demonstrated no correlation with lesion size, a well-known and intuitive result given that very large lesions, while causing many global deficits can spare motor function if the corticospinal tract is uninvolved whereas a very small lesion of the internal capsule can lead to major motor deficits. Our measure of maximally disconnected subgraph size captures this important distinction. We find that grip strength has a strong inverse correlation (*R = −0.62*) with *k*_*optimal*_.

**Table 1:** Spearman correlation of disconnected subgraph size and lesion size to clinical stroke measures. Lesion size and disconnected subgraph size show an identical relation with initial NIHSS admission score. However, lesion size has no correlation with relative grip strength while disconnected subgraph size shows a strong inverse correlation of −0.62.

	NIHSS admission		Relative grip strength	
	*R*	*p-value*	*R*	*p-value*
Lesion size	0.63	0.038	−0.009	0.98
Disconnected subgraph size	0.63	0.039	−0.62	0.040

## 4 Discussion

We present a set of fast and accurate approaches for estimating the overall connectivity loss matrix and maximally disconnected subgraph using shortest path tractography in white matter voxel graphs. There are multiple potential benefits of using a white matter voxel graph to construct a connectivity matrix based on shortest paths compared to streamline based measures. First, Dijkstra's shortest path algorithm is guaranteed to find a path between voxels in different regions no matter their spatial distance. Secondly, the paths connecting two regions are weighted by the geometric means of the edge probabilities instead of each being treated as equally likely as is typically done with streamline counts. Consequently, shortest path estimations have high sensitivity and can capture small reductions of connectivity between many pairs of regions which is evident in Fig. 3, providing unique disconnection information even when lesions are similar in size and location. For connectivity matrices constructed with streamline tractography, many region pairs that should be connected appear disconnected due to length and crossing fiber effects that ultimately lead to premature streamline termination and attempts to overcome this by growing more streamlines leads to many false connections or by dilating gray matter region labels deeper into white matter leads to inaccurate streamline labelling. Although weights can be generated for streamlines using approaches such as SIFT2 or COMMIT for either pruning false streamlines or creating weighted connectivity loss matrices, they do not address the length and crossing fiber effects that lead to many disconnected region pairs that should be connected (Smith et al., 2015 & Daducci et al., 2015). Consequently, the accurate estimation of the true underlying disconnectome or percent connectivity loss remains difficult with streamline approaches. A disadvantage of our shortest path approach is that it also contains many false positive connections as with streamline tractography. However, it's guaranteed to contain all the true positive connections as well which streamline approaches cannot guarantee. Although not investigated here, the weights of each shortest path could be used for pruning out false positive paths and approaches such as COMMIT and LiFE could also be integrated for pruning false positive paths (Daducci et al., 2015 & Pestilli et al., 2014). Moreover, shortest path measures are computationally costly: calculating 2-billion+ shortest path connections between all voxels at the gray-white matter interface per brain can be impractical. We therefore introduce a key strategy to overcome this computational burden: subsets of interface nodes are uniformly sampled at the gray-white interface. In the Appendix we show that the construction of shortest path probability weighted structural networks can be estimated accurately and quickly by only finding shortest paths from uniformly sampled sets of gray-white interface voxels. The subsampled structural network has a Pearson *R*-score of 0.99 with its full counterpart. With this, the task of computing shortest paths decreases from ~24 h to minutes per subject.

With this accelerated estimate of shortest paths, it becomes practical to derive weighted estimates of the % loss of connectivity across all regions pairs when a patient's lesion is projected into a diffusion data set obtained from a healthy brain at high spatial and angular resolution. To make this robust at the population level, it is straightforward to take the same lesion and project it into many normal diffusion data sets. Here, we used 210 of the normal unrelated HCP adult subjects and find that our results with them provide remarkably consistent and clinically plausible maps of disconnection. A decided advantage of this approach is that a weighted matrix of lost connections over the entire brain can be readily generated for individual patients, without the need for a lengthy and clinically impractical diffusion tensor scan from the same patient. All that is needed is a reliable segmentation of the patient's lesion, obtained from routine clinical MRI. It is important to note that our approach is not suitable for patients if white matter reorganization is suspected. Likewise, our approach is not suitable in longitudinal studies.

A second major goal of our work was to develop an algorithm that could consistently reduce the size of the overall connectivity loss matrix because visualizing the full disconnectome is cumbersome and can be difficult to interpret. Rather than a simple arbitrary threshold approach, we developed an algorithm that quantifies the extent of the cortical disconnection network and extracts the most disconnected set of connections and cortical regions from the connectivity loss matrix, i.e., the disconnectome input that share disconnectivity. This is illustrated in Fig. 7 where the full disconnectome in the top panel containing a majority of connections with small losses in connectivity is entered into our algorithm where primarily regions and connections experiencing a large percent connectivity loss are extracted to form the maximally disconnected subgraph in the bottom panel. To do this, we extended the *k*-heaviest subgraph problem by defining an optimal number of nodes, *k*_*optimal*_, to grow the heaviest subgraph. As shown in the results, the growth profiles of the magnitude of the change in the weight of the subgraph for each *k*th node that is added have concave shapes with clearly defined maximums. We define the subgraph where the maximum occurs as the maximally disconnected subgraph. The maximally disconnected subgraph is typically not a maximally disconnected clique because our algorithm does not require every node to be adjacent. This requirement is relaxed because enforcing every node to be a neighbor inherently overrides the goal of extracting the heaviest subgraph because a clique of *k* nodes is not guaranteed to be the *k*-heaviest subgraph. *k*_*optimal*_ can be robustly estimated from 100 or more subjects for patients with a standard deviation ≤1. Here too, there is a significant computational cost to estimating maximally disconnected subgraphs. This motivated our introduction of a greedy algorithm to accelerate this process. In the appendix, we demonstrate that our greedy algorithm closely estimates the full approach (*R = 0.99*).

Neurobiologically, the set of nodes in the disconnected subgraphs up to *k*_*optimal*_ share the cumulative greatest loss in connectivity due to the lesion to each other. The maximally disconnected subgraph finds the set of brain regions that are maximally disconnected from each other due to a lesion. After *k*_*optimal*_, further nodes added do not share as great of a connectivity loss due to the lesion to all the other regions present in the disconnected subgraph. In our results, we focus the use of our algorithm on individual patients, rather than large sample averages to make it evident that our algorithm is specifically extracting just those cortical regions and connections between them with a shared large connectivity loss. Moreover, with the results from our simulated lesions in Fig. 8 it becomes clear that the maximum number of nodes *k* can change dramatically as a function of small shifts in lesion location, independent of lesion size. This is governed by how many unique connections pass through a given location and how many connections for a given pair intersect it. Once a maximally disconnected subgraph is generated, the impact on an individual patient can be readily visualized and direct clinical correlation becomes feasible. For example, the disconnection subgraphs shown in Fig. 5 sit on top of known motor regions: precentral, postcentral, brainstem, pallidum, and thalamus, consistent with the primary clinical deficit in patients 1 and 2: unilateral hemiparesis.

In parallel, disconnection subgraphs from many patients can be used for correlation with clinical signs (as we demonstrate with grip strength measures) or intersected to find common underlying structural abnormalities for a population with similar deficits. For example, the core disconnection of motor regions and corticospinal tract shown in Fig. 6 is not surprising given the selection of patients with hemiparesis. Although *k*_*optimal*_ correlates with the relative grip strength of stroke patients while lesion size doesn't, *k*_*optimal*_ is not specific to the motor network. It is possible for patients to have a high *k*_*optimal*_ without a grip strength deficit. Because the subset of patients used in our study have motor deficits, *k*_*optimal*_ correlated with the relative grip strength. Consequently, *k*_*optimal*_ should only be used for correlation on patients with similar deficits.

Most disconnection studies do not assume a linear association between the number of affected tracts and functioning, because the number of tracts varies considerably as a function of the region pair size and algorithmic parameters that may differ considerably between different analyses and thus yield highly variable results. Consequently, the number of tracts is not a functionally meaningful metric. Instead, the relative loss of tracts between pairs of regions or relative regional loss or relative tract volume lost are assessed. For such relative estimates, one may expect that higher percentages of connections lost due to intersecting a larger lesion, will results in more severe functional impairments with less remaining intact tracts being able to functionally compensate for the white matter damage.

While the impact of discrete lesions on the connectivity of multiple critical white matter pathways on complex behavior has been shown previously, this has largely been accomplished by mapping local predefined sets of white matter tracts with streamlines (Rusconi et al., 2009). Preselection of tracts always introduces a bias and there is potential to miss important disconnections that are outside of this predefined search space that could be treatment targets.

Disconnection can also be quantified without preselecting sets of tracts. However, prior studies that avoided preselection have had marked methodological disadvantages. For example, Kuceyeski et al., 2013 only quantified the percent disconnection at the region level which doesn't elucidate what additional regions that region is disconnected from. Although Langen et al., 2018, measured the percentage of disconnection at the region-to-region level, they used DTI and tracked through lesioned tissue with distorted tensor orientations. Consequently, the estimation of the disconnectome is less accurate because of the inability to accurately track through regions with multiple fiber populations. Moreover, performing streamline tractography on distorted diffusion information in lesioned tissue is known to accumulate into large errors which accumulate into connectomes and consequently disconnectomes (Greene et al., 2018).

In summary our method provides a fast, accurate, robust and unbiased approach for studying the impact of lesions and location on the entire connectome that does not rely on streamline tractography and avoids tracking through lesioned white matter tissue. All that is required is a well segmented lesion. Moreover, it makes visualization and clinical correlation of the disconnection tenable and feasible. Using the provided methodology on a large dataset of behaviorally well-characterized stroke patients in the future may help to further our functional understanding of specific aspects of the structural connectome. Similarly, our approach may help to identify aspects of connectivity indicative of the potential for recovery on the level of individual patients to individualize therapy and improve outcome in stroke patients in the future. Our software package for performing shortest path tractography, constructing connectomes and disconnectomes, and finding maximally disconnected subgraphs can be found online at http://github.com/clintg6/ShortestPathTools.

## Figures and Tables

**Fig. 9:**
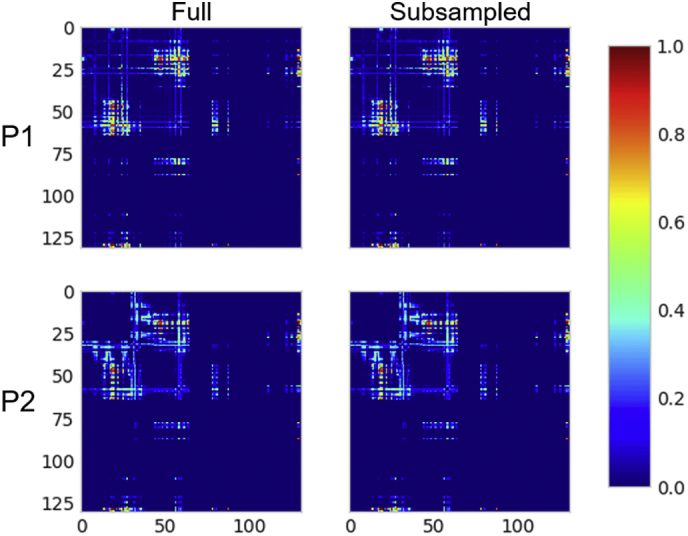
On the left connectivity loss matrices estimated from shortest paths found for the full set of combinations of interface voxel pairs for two patients. On the right are connectivity loss matrices estimated from shortest paths found for only a subsampled set of interface voxel pairs. The matrices in the left and right columns are virtually indistinguishable from each other in the same patient. The Spearman correlation between the full and subsampled connectivity loss matrices for both patients is 0.99.

**Fig. 10:**
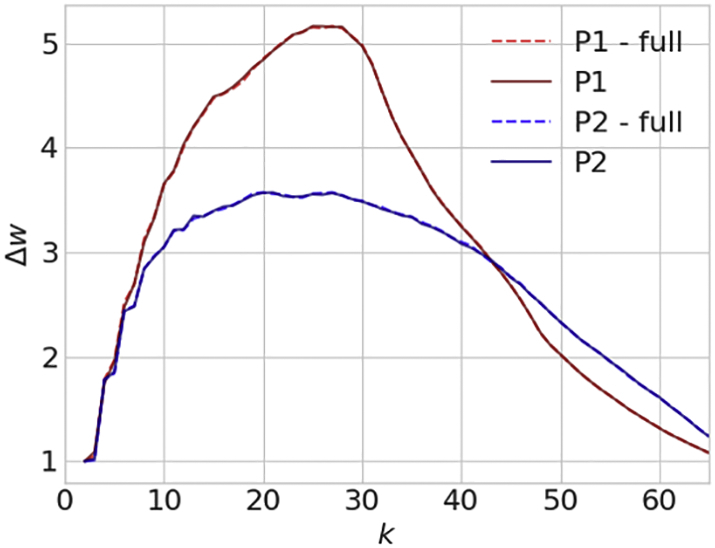
Change in the weight of the *k*-heaviest subgraph at each iteration of *k*. profiles are plotted for both patients from full and subsampled constructed connectivity matrices. The full and subsampled profiles in each patient have a concave structure and are again virtually indistinguishable from each other. In both patients, the full and subsampled profiles have a Spearman correlation coefficient of 0.99 and peak at the same *k*_*optimal*_.

**Fig. 11:**
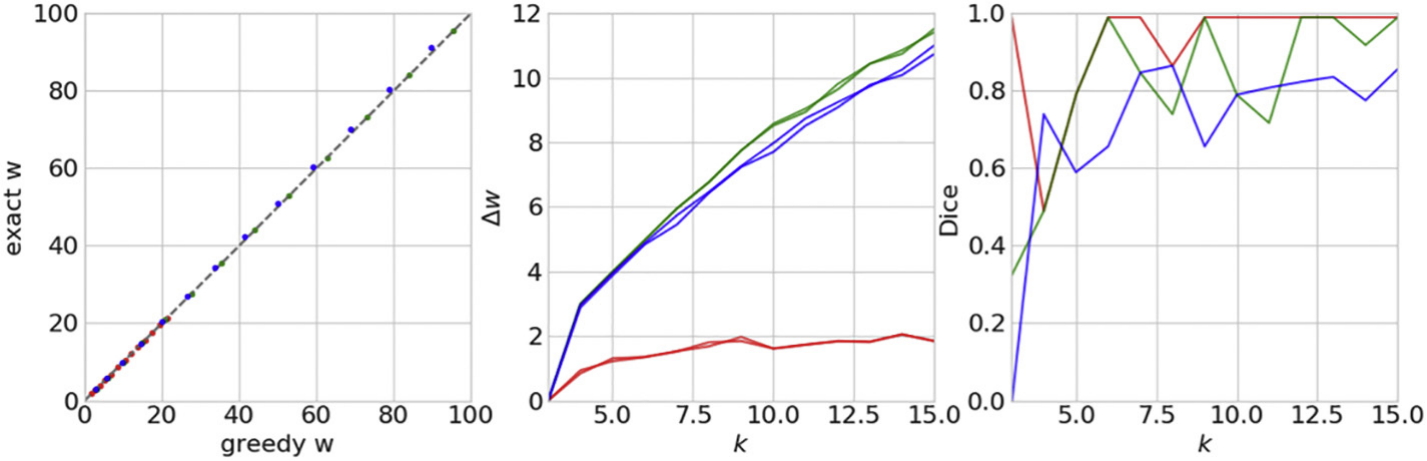
On the left the weight of the subgraph for each iteration of *k* up to *k* = 15 is plotted for the greedy solution and the exact solution for three stroke patients. Patient 3 (red) weights do not reach as large of a weight at *k* = 15 compared to Patient 4 (green) and Patient 5 (blue). Patient 3's lesion is only 132 voxels compared to the large lesions of Patient 4's at 8498 voxels and Patient 5's at 5624 voxels. Unity dashes line is in gray. The change in the total edge weight of the subgraph as it grows in the number of nodes *k* for our greedy approach and exact solution grows larger for larger lesions Patient's 4 & 5 compared to small lesions like with Patient 3. The dice overlap for the set of nodes from the exact subgraph and our greedy subgraph for each iteration of *k* is plotted on the right. Initially, the agreement is poor but as *k* increases the agreement is excellent. The dice overlap for Patient 3 and Patient 4 is 1.0 at *k* = 15 and 0.87 for Patient 5.

## Appendix A Validation data

### A.1 Subsampling validation

In Fig. 9, Connectivity loss matrices estimated from shortest paths found for the full set of combinations of interface voxel pairs are plotted in the left column for two patients. On the right connectivity loss matrices estimated from shortest paths found for only a subsampled set of combinations of interface voxel pairs are plotted. The matrices in the left and right columns are virtually indistinguishable for both patients. The Spearman correlation coefficient for both patients between the full and subsample connectivity loss matrices is 0.99.

In Fig. 10, the profiles for the change in the weight of the *k*-heaviest subgraph at each iteration of k are plotted for both patients from full and subsampled constructed connectivity matrices. The full and subsampled profiles in each patient have a concave structure and are again virtually indistinguishable. In both patients, the full and subsampled profiles have a Spearman correlation coefficient of 0.99. Both sets of profiles for both patients peak at the same *k*_*optimal*_.

### A.2 Greedy algorithm validation

To measure the performance of our greedy algorithm compared to the exact solution, the weight of the subgraph is plotted for each iteration of *k* up to *k = 15* for the greedy and exact solutions from connectivity loss matrices from three patients' stroke lesions in Fig. 11 on the left. The solutions agree excellently with a Spearman *R-score* of 0.99. Patient 3 (red) weights do not reach as large of a weight at *k* = 15 compared to Patient 4 (green) and Patient 5 (blue). Patient 3's lesion is only 132 voxels compared to the large lesions of Patient 4's at 8498 voxels and Patient 5's at 5624 voxels. The agreement between the solutions is also seen in the middle plot for the change in the weight of the subgraph for each node *k* added to the subgraph. Again Patient 4's and Patient 5's change in weight curves grow larger for each node added due to larger lesion size compared to Patient 3's. On the right in Fig. 11, the dice overlap between the nodes making up the exact heaviest subgraph of *k* nodes and the greedy heaviest subgraph (Dice, 1945). At *k* = 15, Patient's 3 & 4 both have a dice overlap of 1.0 i.e. perfect agreement between the exact solution and greedy solution. For Patient 5, the dice overlap is 0.87. As *k* increases, the dice overlap grows. This suggests that there is minor disagreement initially about which node to add but the greedy solution converges as *k* increases to the exact solution.
